# Annotations

**Published:** 1903-01-24

**Authors:** 


### ANNOTATIONS.

### The London Hospital Appeal.

THE forcible appeal on behalf of the London Hospital which was issued last week by the Hon. Sydney Holland will, we are sure, meet with a hearty response. The City of London has always been famous for its charities and for the readiness with which its citizens have supported those deserving institutions which abound in its midst, and we imagine that no appeal could be more worthy of support than this which is now being made for the London Hospital. Whether we regard the extent of the good work done, the extreme urgency of the cases treated, the vastness of the population who trust to the hospital for that high-class medical and surgical assistance which is quite unattainable in their own homes, or to the energy and businesslike manner in which the affairs of the charity are managed, the London Hospital stands forth as an institution which not only demands but thoroughly deserves the most liberal support. We especially urge the claims of this great establishment for succour of the sick and the poor, because the London Hospital, although no doubt a sturdy and most convincing beggar, is not a constant or an irritating one. Every year, doubtless, as indeed every day, something is wanted, and the statement of such a want often brings a response; but the system of only once in five years making a strong and general appeal, which was introduced by Mr. John Henry Buxton, late treasurer of the hospital, is one which is worthy of every encouragement and deserves well of philanthropists. It is, indeed, one which should be specially attractive to business men. The mere exercise of choice between the conflicting claims of many charities is a trouble and an embarrassment to many, and the London Hospital does well, and deserves hearty support, in making only a quinquennial appeal. Let us trust that the efforts to be made this year in favour of what certainly is London's greatest hospital, if not perhaps its greatest charity, will draw money from the pockets of all classes; and let us not forget, when embarrassed by the many distressful objects which come upon us, that in the words of our genial contemporary, *Mr. Punch*, the London Hospital has "the greater need."

### The Limits of Gynæcology.

IN delivering his valedictory address on retiring from the presidency of the British Gynæcological Society, Sir J. Halliday Croom discussed the future of gynæcology regarded as a specialty, pointing out how difficult it was becoming to draw the line between gynæcological and general surgery. The matter is one of considerable interest, for so strongly of late has this department drifted towards surgery pure and simple that it is becoming by no means clear why it should any longer be regarded as a specialty. Naturally, as is the case with breasts and tongues and other regions of the body, certain surgeons take up and become more or less famous for their treatment of the one or the other, and in this sense the treatment of all these various organs may be regarded as specialties; but they do, in fact, remain mere branches of general surgery and are practised by general surgeons. Gynæcology, however, professes to stand apart. It has grown up and developed from that old custom which, at a time when diseases of women were regarded as internal and inaccessible, handed over their treatment to the obstetric physician, and thus it has happened that, notwithstanding the surgical tendencies which have marked almost every stage of its development, it has remained in the hands of special officers and by a purely artificial system of hospital appointments has been more or less divorced from surgery. The tendency is to ignore the medical element in gynæcology and to put midwifery on one side unless it can be diverted into surgical grooves, as in the modern resuscitation of Cæsarian section; and that being so it may be questioned whether there is any reasonable excuse for the continued recognition of gynæcology as being any more a specialty than are half a dozen other departments of general surgery.

### Baby Farming.

THE case of the two wretched women who lie under sentence of death for child murder will doubtless be taken by some as illustrating the old adage that "murder will out," and as giving such proof of the risks of dabbling in this horrible traffic as to afford some assurance that it will cease. We think otherwise. What this trial has done has been to lay bare the details of an individual case in a widespread system which is in constant operation in our midst for the destruction of inconvenient babies, and it showed well what a chapter of accidents and what recklessness on the part of the criminals were necessary to bring even such atrocious crimes as these to light. The fact is that as the law at present stands the facilities which exist for doing to death those unfortunate infants whose mothers find it worth their while to get rid of them, are such that we can hardly hope that the detection and punishment of two peculiarly clumsy professors of the art will have much effect in putting a stop to the practice. The weak spots in the law are those which have to do with the "one-child" and the "lump-sum" cases. There seems nothing to prevent the receiver of an infant paid for in a lump sum, conforming with the law by giving notice, and then straightways defeating its action by removing with the baby into another district where she can quietly get rid of it unmolested by the visits of inspectors; while the receiver of the single child is under even greater temptation and far less under the control of public opinion than was the "baby farmer" of old. No woman could farm a number of children without the matter becoming known, especially if funerals were frequent. But in view of the constant shifting of the population the single case is very difficult to trace, while the temptation to murder—for murder it is even though it be done by means of patent foods—is greatly increased by the fact that till number one is got rid of number two—with its premium—cannot be received. Reform of the law is needed in the direction of enforcing the continued registration (after the manner of the ticket-of-leave man) of the receiver of a "lump sum case," and the application to the reception of a single child of all those precautionary safeguards which are already applied to the case where more than one child is received.

